# The influence of emotion regulation on convergent thinking under different cognitive loads

**DOI:** 10.1371/journal.pone.0353216

**Published:** 2026-08-13

**Authors:** Yuan Zhao, Yue Wang, Feng Lu, Jiajing Wang, Qiqi Xing, Walton Wider

**Affiliations:** 1 Police Officer Academy, Shandong University of Political Science and Law, Jinan, Shandong, China; 2 Faculty of Education and Liberal Arts, INTI International University, Nilai, Negeri Sembilan, Malaysia; 3 School of Education Science, Taizhou University, Taizhou, Jiangsu, China; 4 Journal Editorial Department, Nanjing Normal University of Special Education, Nanjing, Jiangsu, China; 5 Faculty of Business and Communications, INTI International University, Nilai, Negeri Sembilan, Malaysia; Fakultet za pravne i poslovne studije dr Lazar Vrkatic, SERBIA

## Abstract

Convergent thinking is a critical aspect of creative thinking, and it has a substantial influence on the learning and work of college students. Emotion regulation not only enhances emotional well-being but also plays a vital role in the creative process. Additionally, various social and cognitive factors contribute to the completion of complex convergent thinking tasks, with cognitive load being one of the primary determinants. This study recruited 93 undergraduates as participants and randomly divided them into three groups: cognitive reappraisal, expressive suppression, and free viewing. All participants were required to complete a convergent creativity test (Chinese compound remote associate problems, CCRA) under high and low cognitive load conditions. The results showed a significant main effect of cognitive load on convergent thinking scores, with participants achieving higher scores under low cognitive load than under high cognitive load. However, the interaction between cognitive load and emotion regulation strategy on convergent thinking scores was not significant. Response time analyses revealed a significant interaction between cognitive load and regulation strategy: under high cognitive load, cognitive reappraisal was associated with faster responses than free viewing, whereas under low cognitive load, expressive suppression led to faster responses than both cognitive reappraisal and free viewing. This study explored the impact of emotion regulation on convergent thinking under different cognitive loads, which has certain implications for the optimization of college students’ mental and physical health and academic performance.

## Introduction

Creativity has been recognized as an important indicator of individual development and career success since the 1950s [1]. It is a distinguishing human trait, considered one of the most advanced cognitive activities of the human brain [2]. Creativity is typically bifurcated into two types: convergent thinking and divergent thinking [3–6]. Divergent thinking pertains to the ability of individuals to generate novel and diverse outputs from existing information, often from a unique perspective [4,7]. Conversely, convergent thinking employs using acquired knowledge and experience, or analyzing and reasoning about a problem, to arrive at the best answer or strategy when faced with known information [3]. Historically, creativity research has primarily centered on divergent thinking and divergent thinking is deemed as the fundamental aspect of creative thinking [5]. However, in recent years, there has been a growing interest in convergent thinking research within the field of creativity, driven by advancements in neuroscience and increased attention to insight research [8].

Emotion regulation not only ameliorates emotions but also plays a pivotal role in the creative process [9]. Emotion regulation refers to the processes by which individuals influence the experience and expression of their emotions [10]. Among the most widely studied strategies are cognitive reappraisal and expressive suppression. Cognitive reappraisal involves altering one’s interpretation of an emotional stimulus to change its emotional impact and is generally considered an adaptive strategy [9]. Expressive suppression, in contrast, entails inhibiting the outward display of emotions after an emotional response has been generated, often increasing cognitive costs and impairing performance on resource-demanding tasks [11]. In laboratory experiments, to compare the effects of different emotion regulation strategies, researchers typically include a control condition known as free viewing, in which participants are instructed to view emotional stimuli without applying any regulation strategy [12]. Free viewing serves as a control condition, allowing participants to process emotional information naturally without deliberate regulation, thereby reflecting baseline emotional responses [13] These strategies differ in cognitive demands and effectiveness, which may have implications for creative performance under varying cognitive loads. In their exploration of emotion regulation and creativity, Zhang, Liu [14] found that cognitive reappraisal enhances individual creativity in the face of task conflicts through the partial mediation of perspective-taking. Cognitive reappraisal is an antecedent-focused emotion regulation strategy, meaning that it acts before an emotional response occurs by changing how a person interprets an emotional event to modify its impact [15]. Through the mediating role of emotional exhaustion, expression inhibition makes individuals display lower levels of creativity when faced with task conflict. In a recent study, Toscano, Giusino [16] found that an individual’s positive reevaluation is the moderating variable of the influence of innovation style on innovation behavior. This implies that creative individuals are more inclined to exhibit innovative behaviors at work due to their ability to actively comprehend work-related situations and events. In terms of the impact of emotion regulation on convergent thinking, Zhao [17] found that regulating positive emotions through cognitive reappraisal facilitates performance, whereas regulating negative emotions tends to hinder it. Positive emotion regulation refers to managing pleasant emotions such as joy or satisfaction, while negative emotion regulation involves handling unpleasant emotions such as anxiety or anger. This aligns with evidence that positive affect can promote insight and flexible problem-solving by enhancing cognitive flexibility and associative processing [18]. Cognitive reappraisal, by reframing emotional stimuli, may help sustain such positive affect and maintain access to broader associative networks, which are beneficial for convergent thinking. In contrast, regulating negative emotions—especially those involving anxiety—can consume limited cognitive resources and narrow attentional focus [19], potentially reducing the ability to integrate disparate pieces of information into a single correct solution. Furthermore, experimental work has shown that the effects of emotion regulation on creativity depend on both the valence and intensity of the emotion being regulated, as well as the cognitive demands of the task [20]. These findings suggest that the interplay between emotional valence, regulation strategy, and cognitive resource availability is critical in determining convergent thinking performance.

In daily study and work, people often need to undertake complex creative tasks. The completion of complex creative tasks is influenced by various social and cognitive factors, among which cognitive load plays a significant role [21–23]. Cognitive load refers to a psychological condition characterized by an overwhelming influx of information, resulting in a deficiency of psychological resources necessary for problem-solving or task completion, thereby imposing a significant burden on the cognitive system [22]. Recent research has indicated that sustained executive attention and attention-restorative can support executive functioning, inhibit irrelevant distractions, and maintain information integration, thereby facilitating creative problem solving and insight generation [24]. On the contrary, mental distractions and highly distracting work weaken individual creative performance [25]. People often encounter a large amount of information in their work and studies. For college students, with the development of the Internet and the increase of academic pressure, their cognitive load in learning activities may also increase. Consequently, researchers have started exploring ways to optimize learning tasks and improve college students’ learning outcomes. For instance, Gu [26] found that intrinsic cognitive load during foreign language learning decreases as proficiency improves. This suggests that domain expertise can free up cognitive resources for higher-order processing—a principle that may also apply to creativity-related tasks, including convergent thinking, where reduced task-related cognitive load allows more resources to be allocated to integrating information and generating accurate solutions.

The inherent complexity of creative tasks already contributes to cognitive load, and any additional load could further impair performance [21]. However, previous studies on cognitive load and creativity mainly explored the impact of high or low cognitive load on divergent thinking [25,27]. For example, Rodet [25], through laboratory experiments on divergent thinking tasks, found that cognitive load caused by digital memory tasks significantly reduced both the number and diversity of ideas generated during divergent thinking. Similarly, through an experimental study of 151 college students, Huang, Song [27] found that in low cognitive load tasks, mind wandering can promote individual performance in divergent thinking tasks. The reason why most studies focused on the impact of cognitive load on divergent thinking is that Guilford [5] proposed divergent thinking as the core of creative thinking, which has attracted extensive attention from many scholars. However, given that college students are in a critical period for developing higher-order cognitive abilities while often facing substantial academic demands and information overload, it is necessary to explore the impact of cognitive load on their convergent thinking.

Previous studies have demonstrated that emotion regulation strategies influence convergent thinking performance. For instance, Zhao [17] reported that regulating positive emotions through cognitive reappraisal facilitates convergent thinking, whereas regulating negative emotions tends to hinder performance. Similarly, Niermeyer, Ziemnik [28] found that expressive suppression reduces flexibility in problem-solving, thereby impairing convergent thinking accuracy. Building on these findings, it is worth noting that the effects of emotion regulation vary under different cognitive loads. The theoretical link between emotion regulation and convergent thinking can be understood within the framework of Gross’s process model of emotion regulation, which emphasizes the cognitive demands of regulatory strategies [10]. Cognitive reappraisal requires substantial engagement of prefrontal regions to reinterpret emotional stimuli, thereby drawing on working memory and attentional control resources that are also essential for convergent thinking tasks [29]. Similarly, expressive suppression imposes additional physiological and cognitive costs by inhibiting behavioral responses, which can further deplete cognitive resources needed for problem-solving and flexibility [11]. Since convergent thinking relies heavily on executive functions such as controlled attention and working memory [30], any reduction in available cognitive resources due to emotion regulation may directly impair performance on tasks like the Compound Remote Associates Test (CCRA). Empirical evidence supports this theoretical perspective, showing that the effectiveness of emotion regulation strategies is constrained by cognitive resource availability.

In addition, prior research has examined the relationship between discrete emotions such as anger and anxiety and creative performance, including convergent thinking. Anger and anxiety were chosen because they are the most commonly experienced negative emotions among college students in academic and social contexts, such as examinations, public speaking, and interpersonal conflicts [31,32]. Both emotions are high-arousal negative states, which have been shown to exert significant influence on cognitive control and creative performance [33,34]. Compared to sadness or fear, which are typically associated with low approach motivation or avoidance behaviors, anger and anxiety are more likely to interact with cognitive resource allocation during problem-solving tasks [35]. Empirical evidence suggests that anger, as a high-approach motivational state, can enhance persistence and selective attention, potentially facilitating narrowing down to a single correct solution in convergent thinking tasks [36]. In contrast, anxiety is often associated with heightened vigilance and threat-related processing, which can consume working memory resources and impair the integration of information needed for convergent thinking [37]. This pattern suggests that anger and anxiety may exert distinct influences on the cognitive processes underpinning convergent thinking, making them theoretically relevant for investigating the interaction between emotion regulation and cognitive load.

Building on these distinctions between anger and anxiety, it is also important to consider how emotion regulation strategies targeting these emotions operate under varying cognitive load conditions, as cognitive resources play a crucial role in determining their effectiveness. For instance, Gan, Yang [38] employed a dual-task paradigm, in which participants perform two tasks simultaneously to manipulate cognitive load, to investigate how cognitive reappraisal operates under different cognitive loads. In the low-load condition, they found that the amplitude of emotion-enhanced late positive potential (LPP) was significantly decreased by neutral reappraisal compared to negative reappraisal. Under the high-load condition, the regulatory effect of reappraisal disappears. This suggests that successful cognitive reappraisal depends on cognitive resources and working memory processes. If concurrent memory tasks excessively consume the resources necessary to participate in cognitive reappraisal, the effect of emotion regulation will be impaired. Emotion is considered to be a source of external cognitive load, and regulating emotion may lead to the allocation of cognitive resources outside the task being processed [39]. Therefore, it is necessary to consider the impact of emotion regulation on cognitive resources when solving complex convergent thinking tasks and find the best situation to promote people’s convergent thinking performance.

This study aims to investigate the differences in convergent thinking performance under different cognitive loads and examine the influence of emotion regulation. To provide a theoretical framework for understanding how emotion regulation strategies influence convergent thinking under different cognitive load conditions, the present study draws on the emotion regulation flexibility framework [40,41]. This framework emphasizes that the effectiveness of emotion regulation strategies depends not only on the strategy itself but also on the fit between regulatory strategies and contextual demands [40,42]. In other words, no single strategy is universally adaptive or maladaptive; instead, successful regulation requires flexible selection and implementation of strategies according to situational requirements [43].

From this perspective, cognitive load represents an important contextual factor that determines the availability of cognitive resources during creative problem solving [44]. Cognitive reappraisal, as an antecedent-focused strategy, may require cognitive resources initially but can reduce emotional interference and facilitate adaptive processing [45]. In contrast, expressive suppression may consume fewer resources during implementation but may have different effects depending on task demands [46]. Therefore, the interaction between emotion regulation strategy and cognitive load should be considered when examining convergent thinking performance.

Based on the emotion regulation flexibility framework and previous empirical findings, the following hypotheses were proposed. H1: Both cognitive reappraisal and expressive suppression are expected to reduce self-reported negative emotion ratings following emotional stimuli, with cognitive reappraisal producing a stronger reduction than expressive suppression. H2: Convergent thinking performance will be significantly better under low cognitive load compared to high cognitive load. H3: The effect of emotion regulation strategies on convergent thinking performance will vary by cognitive load. Specifically, cognitive reappraisal is expected to show greater advantages when cognitive resources are more limited (e.g., under high cognitive load), whereas expressive suppression may be more effective when cognitive load is low. The results will offer valuable insights and recommendations for fostering convergent thinking among college students.

## Design and methods

### Participants

A prior power analysis conducted by G*power 3.1.9.7 indicated that a minimum requirement of 66 participants (*α* = 0.05, power [1 – *β*] = 0.95, *η*^*2*^ = 0.25), considering the interaction between cognitive load (high, low) and regulation strategy type (cognitive reappraisal, expressive suppression, and free viewing). A total of 93 undergraduates, including 63 males, were recruited from the online communities at a university in Shandong, China (e.g., WeChat groups, QQ groups) through convenience sampling, the sample size of this experiment conforms to the statistical test standard (93 > 66). The participant recruitment period for this study was from September 10, 2023 to November 8, 2023. The recruitment strategy employed was a rolling recruitment approach, entailing the immediate execution of the corresponding experimental procedures for each successfully recruited participant. Upon the conclusion of the recruitment period, the experimental data collection process was finalized. All participants were native Mandarin Chinese speakers, right-handed, and possessed (corrected) normal vision, with no history of brain injury or mental illness. They voluntarily participated in this experiment, with no prior exposure to similar experiments. The study received ethical approval from the ethics committee of Shandong University of Political Science and Law and was conducted in accordance with the principles outlined in the Declaration of Helsinki. Prior to the experiment, all participants read and acknowledged the informed consent form in writing before completing the questionnaire. They were paid 20 yuan after the experiment and could terminate the experiment at any time if they felt uncomfortable.

### Experiment materials

#### Emotional situation sentences system, ESSS.

The ESSS is mainly used in relevant research on the emotional priming of college students, with good arousal and ecological validity, and is a good tool for studying the emotional problems of college students [47]. For this study, we selected 24 sentences associated with anger, such as “I heard someone whisper bad things about my parents” (average valence:2.44 ± 0.18; average arousal: 7.17 ± 0.09) and 24 sentences associated with anxiety, such as “I’m going to give a speech in front of hundreds of people” (average valence: 2.45 ± 0.35; average arousal: 6.73 ± 0.20) as experimental materials. The sentences were selected based on the standardized ratings provided by the original ESSS, including emotional category, valence, and arousal scores. Items with clear emotional classification and appropriate emotional intensity were chosen to ensure effective emotion induction. As the ESSS has been previously developed and validated as a standardized emotional stimulus system, no additional factor analysis was conducted in the present study. Two anger sentences and three anxiety sentences were selected for the practice phase.

#### Chinese compound remote associate problems, CCRA.

The CCRA is frequently utilized in creativity research as a measure of an individual’s convergent thinking abilities [6,48]. In the CCRA task, participants are presented with three unrelated Chinese characters (e.g., 命 [life/fate], 男 [male], 学 [learning]) and are required to generate a Chinese character (e.g., 生 [life/student]) that can be combined with each of the three characters to form meaningful compound words (e.g., 生命 [life], 男生 [male student], 学生 [student]). The 48 items selected for the main experiment were meticulously balanced in terms of difficulty and response rate, while an additional five items were chosen for the practice phase.

#### The positive and negative affect schedule, PANAS.

To counteract the potential influence of emotions on convergent thinking [6,49], the PANAS was utilized to evaluate participants’ emotional states prior to the experiment [50,51]. The PANAS comprises two subscales: Positive Affect (PA) and Negative Affect (NA). The PA subscale consists of 10 items designed to assess positive emotions, such as interest and enthusiasm, while the NA subscale comprises 10 items aimed at measuring negative emotions, including nervousness and panic. Each item was rated on a 5-point Likert scale, ranging from 1 (strongly disagree) to 5 (strongly agree). In this experiment, the Cronbach’s alpha coefficients of the full scale, PA subscale, and NA subscale were 0.91, 0.89, and 0.91, respectively.

#### Emotion regulation questionnaire, ERQ.

To control the influence of the emotion regulation strategies used by the participants, the ERQ compiled by Gross and John [9] was used to measure the participants’ tendency to use emotion regulation strategies in daily life. The ERQ is divided into two dimensions: cognitive reappraisal and expressive suppression. The cognitive reappraisal scale included 6 items, such as “When I want to feel more positive emotions, I will change my way of thinking about the situation.” The expressive suppression subscale includes 4 items, such as “When I feel negative emotions, I make sure I do not show them.” Each item was rated on a 7-point Likert scale, ranging from 1 (strongly disagree) to 7 (strongly agree). In this experiment, the Cronbach’s alpha coefficients of the full scale, cognitive reappraisal subscale, and expressive suppression subscale were 0.71, 0.86, and 0.64, respectively.

### Experimental procedure

**Fig 1:**
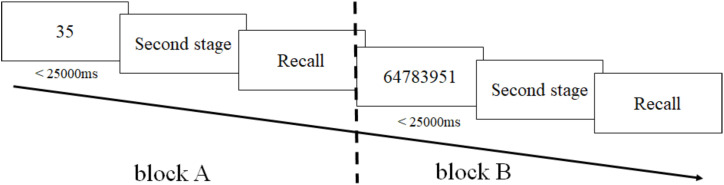
Procedure of this experiment.

When the participants arrived at the laboratory, they were randomly assigned to one of three groups: cognitive reappraisal, expressive suppression, or free viewing. Initially, participants were instructed to complete the PANAS and ERQ. Following this, participants were informed of their right to withdraw from the experiment at any point should they experience discomfort. The experiment was conducted using E-prime 3.0 (Psychology Software Tools, Inc., Sharpsburg, PA, USA) and consisted of two cognitive load blocks (Block A: low load; Block B: high load) arranged in an ABBA sequence to control for order effects (Fig 1). Each block comprised three stages: (1) memory load induction, (2) emotion regulation and convergent thinking task, and (3) recall.

**Stage 1: Memory load induction.** Based on previous studies [52,53], a series of numbers were displayed on the screen during the first stage. In block A, a two-digit number (e.g., 35, with distinct tens and unit digits) was displayed, while in block B, an eight-digit number (e.g., 64783951, with distinct digits) was shown. Whether in block A or B, participants were tasked with memorizing the displayed numbers within a 25-second timeframe and were later prompted to recall them at the experiment’s conclusion. After memorizing the numbers, participants pressed the “SPACE” key to proceed to stage 2.

**Fig 2:**
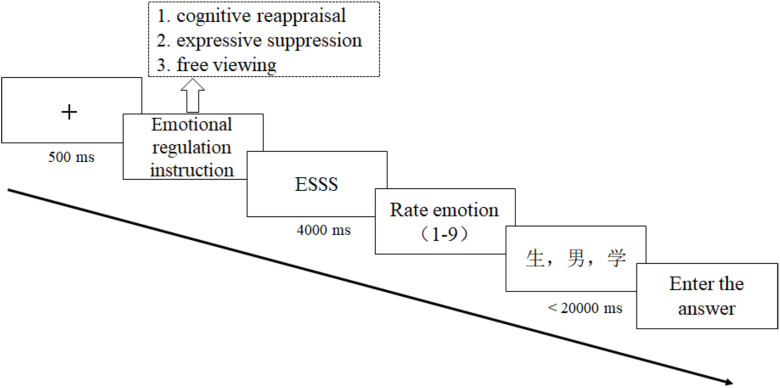
Procedure of stage two.

**Stage 2: Emotion regulation and convergent thinking task.** Participants were instructed to regulate their emotions according to the provided instructions and then complete 12 CCRA items (Fig 2). Each trial commenced with the display of a “+” in the center of the screen for 500 ms, followed by the presentation of specific “emotion regulation instructions” tailored to each participant group (cognitive reappraisal, expressive suppression, free viewing). Based on previous emotion regulation studies conducted in Chinese cultural contexts [54,55], participants in the cognitive reappraisal group were instructed as follows: “Please carefully read the emotional situation sentences while maintaining an objective attitude, imagine that these descriptions are not true, reinterpret the situation, and strive to avoid experiencing any emotions”. Participants in the expressive suppression group were given the following directive: “Please concentrate on the forthcoming emotional situation sentences. While experiencing emotions, abstain from expressing your feelings and endeavor to conceal your emotions effectively, ensuring that others cannot discern your emotional experience”. Participants in the free viewing group were instructed as follows: “Please pay attention to the following sentences”.

The exposure durations for each stimulus presentation were determined based on prior emotion regulation and insight problem-solving paradigms [38,56]. The instructions were displayed for a duration of 1,000 ms. Subsequently, an emotion-inducing sentence from the ESSS was displayed on the screen for a duration of 4,000 ms. Participants needed to use different emotion regulation strategies to regulate the emotions generated after seeing ESSS. Participants were then directed to rate their current emotions on a 9-point scale, with “1” indicating “very unpleasant” and “9” indicating “very pleasant”. Following this, a CCRA item was presented on the screen for 20000 ms. If participants come up with an answer, they immediately press the “SPACE” and input their response in the answer interface. If participants were unable to formulate an answer within the 20000 ms timeframe, this interface would be skipped.

**Stage 3: Recall.** At the end of each block, participants recalled and entered the number memorized in Stage 1.

Participants were allowed to commence the practice phase of the experiment once they comprehended the experimental procedure. The formal experiment could only be conducted once participants fully understood the experimental procedure and the evaluation criteria. If necessary, the practice phase could be repeated until full comprehension was achieved. Following the completion of the experiment, a validity test was conducted to assess the effectiveness of the emotion regulation strategy. Participants were prompted to rate “the extent to which you successfully adjusted emotions elicited by the emotional situation sentences through the emotion regulation strategy” on a scale of 1 (strongly low) to 9 (strongly high). For the free viewing group, participants were asked a yes/no self-report question after the experiment:“Did you refrain from using any emotion regulation strategies throughout the experiment when exposed to the emotional situation sentences?”

### Data analysis

Data analyses were performed using IBM SPSS Statistics (Version 26; IBM Corp., Armonk, NY, USA).

One-way analyses of variance (ANOVAs) were conducted to examine group differences in control variables, including Positive Affect (PA), Negative Affect (NA), and habitual emotion regulation strategies measured by the ERQ. To test the main hypotheses, mixed-design ANOVAs were conducted, with cognitive load (low vs. high) as a within-subject factor and emotion regulation strategy (cognitive reappraisal, expressive suppression, free viewing) as a between-subject factor. Accuracy and response time on the CCRA task were used as dependent variables. For analyses involving emotion type, separate 3 (emotion regulation strategy) × 2 (emotion type) mixed-design ANOVAs were conducted under high and low cognitive load conditions. These analyses were conducted separately to examine strategy and emotion effects within each level of cognitive load. Simple main effects analyses with Bonferroni-adjusted post hoc comparisons were conducted to further examine significant interaction effects. Participants with four or more errors in the high-load memory recall task were excluded from further analyses.

Effect sizes were reported as partial eta squared (*η²*) for ANOVA effects and Cohen’s d for pairwise comparisons. According to conventional criteria, *η²* values of 0.01, 0.06, and 0.14 represent small, medium, and large effects, respectively, and *Cohen’s d* values of 0.20, 0.50, and 0.80 indicate small, medium, and large effects [57,58].

## Results

### Analysis of control variables

A one-way analysis of variance (ANOVA) was used to analyze the control variables. As depicted in Table 1, no significant differences were observed in the PANAS (PA and NA: *F*_(2, 91)_ = 0.44, *p* = .647; *F*_(2, 91)_ = 1.85, *p* = .163) and ERQ across the three groups (*F*_(2, 91)_ = 0.56, *p* = .574; *F*_(2, 91)_ = 0.69, *p* = .507). Consequently, baseline differences in affect and habitual emotion regulation tendencies are unlikely to account for the observed experimental effects.

**Table 1:** Analysis of control variables.

Emotion regulation strategy	PANAS		ERQ	
	PA	NA	Cognitive reappraisal	Expressive suppression
**Cognitive reappraisal (*n* = 32)**	29.63 ± 8.04	22.50 ± 8.09	5.02 ± 0.99	4.10 ± 0.98
**Expressive suppression (*n* = 31)**	27.97 ± 6.83	19.19 ± 7.49	4.88 ± 1.13	3.79 ± 1.21
**Free viewing (*n* = 29)**	29.48 ± 7.68	22.20 ± 6.76	4.74 ± 1.04	3.92 ± 0.87
***F***	0.44	1.85	0.56	0.69
***η*** ^***2***^	0.01	0.04	0.01	0.02

### Analysis of the effects of emotional regulation

After the experiment, participants completed a validity test for the operation of emotion regulation strategies. The manipulation check indicated that participants in the cognitive reappraisal and expressive suppression groups reported using the instructed emotion regulation strategies, respectively. Both groups scored significantly higher than the random level, which refers to the midpoint (5) of the 9-point rating scale used in the test (7.00 > 5, 7.06 > 5), with no significant difference between the two groups (*t*_(61)_ = −0.26, *p* > .05, *d* = −0.06). Furthermore, all par*t*icipants in the free viewing group reported that they had not intentionally used any emotion regulation strategies when reading the emotional situation sentences.

As shown in Table 2, under high cognitive load, the effect of emotion regulation strategy on anger was significant, *F*_(2, 89)_ = 5.06, *p* = .008, *η²* = .10, whereas the effect on anxiety was not significant, *F*_(2, 89)_ = 2.54, *p* = .084, *η²* = .05. Under low cognitive load, the effect of emotion regulation strategy on anger was also significant, *F*_(2, 89)_ = 4.34, *p* = .016, *η²* = .09, while the effect on anxiety remained non-significant, *F*_(2, 89)_ = 1.58, *p* = .212, *η²* = .03. Overall, across both high and low cognitive load conditions, emotion ratings followed the trend of cognitive reappraisal > expressive suppression > free viewing. These results indicate that both classical emotion regulation strategies effectively alleviated negative emotions, with cognitive reappraisal showing a more pronounced regulatory effect, thus supporting H1.

**Table 2:** Analysis of the effects of emotional regulation.

Emotion regulation strategy	High		Low	
	Anger	Anxiety	Anger	Anxiety
**Cognitive reappraisal (*n* = 32)**	2.48 ± 0.59	2.44 ± 0.69	3.05 ± 0.67	2.95 ± 0.77
**Expressive suppression (*n* = 31)**	2.22 ± 0.96	2.14 ± 1.01	2.66 ± 0.97	2.74 ± 1.02
**Free viewing (*n* = 29)**	1.89 ± 0.50	2.00 ± 0.61	2.47 ± 0.70	2.55 ± 0.80
***F***	5.06^**^	2.54	4.34^*^	1.58
***η*** ^***2***^	0.10	0.05	0.09	0.03

### The effect of emotion regulation strategies on convergent thinking under different cognitive loads

Numerous seminal studies suggest that in tasks involving cognitive load, a higher error count in number recall indicates that participants did not sustain a high cognitive load during task execution [53,59]. Conversely, fewer errors imply that participants remembered the numbers during the task, albeit not with complete accuracy [53,59]. Following the protocol of Gilbert and Hixon [59], this experiment excluded participants who had a recall error count of 4 or more under high cognitive load. The final sample comprised 78 participants, whose data were processed based on combined performance across both blocks A and B: 29 in the cognitive reappraisal group with an effective rate of 90.63%, among which 10 made minor errors (error count less than 2) and 19 had perfect recall; 23 in the expressive suppression group with an effective rate of 74.19%, among which 7 made minor errors (error count less than 2) and 16 had perfect recall; 26 in the free viewing group with an effective rate of 89.66%, among which 8 made minor errors (error count less than 2) and 16 had perfect recall.

**Fig 3:**
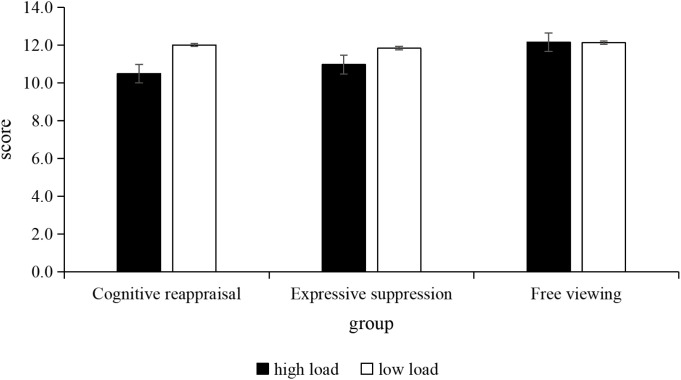
The effects of emotion regulation strategy type and cognitive load on CCRA score. *Note.* Bars represent group means for each regulation strategy under different cognitive load conditions. Error bars represent standard errors of the mean (*± SE*).

A 2 (cognitive load: high, low) × 3 (regulation strategy type: cognitive reappraisal, expressive suppression, and free viewing) mixed-design ANOVA was conducted to analyze the effects of different regulation strategy types on convergent thinking tasks under different cognitive loads. The main effect of cognitive load on scores was significant (*F*_(1, 75)_ = 3.95, *p =* .05, *η*^2^ = .05), with scores significantly higher under low cognitive load compared to high cognitive load (*MD* = 0.81, *SE* = 0.39, *p =* .044). This result supports H2, indicating that participants performed better on convergent thinking tasks when cognitive load was low (see Fig 3). However, the interaction between cognitive load and regulation strategy type was not significant (*F*_(1, 75)_ = 1.39, *p =* .256, *η*^2^ = .04). Therefore, H3 was not supported for convergent thinking scores, suggesting that the influence of emotion regulation strategies on task accuracy did not significantly differ between high and low cognitive load conditions.

**Fig 4:**
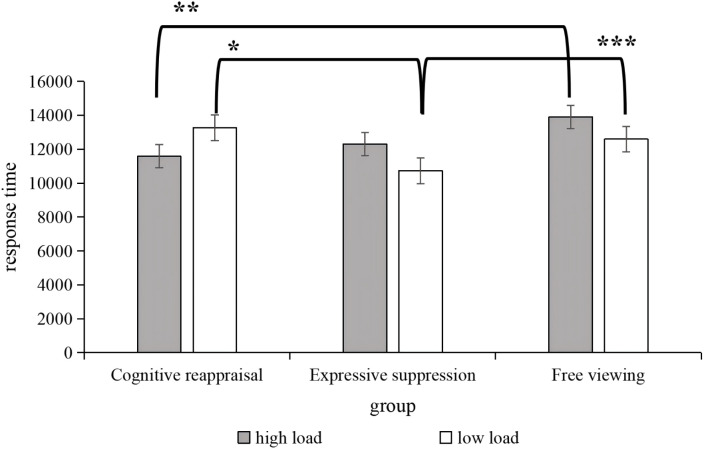
The effects of emotion regulation strategy type and cognitive load on response time. *Note.* Bars represent mean response time for each regulation strategy under high and low cognitive load conditions. Error bars represent standard errors of the mean (*± SE*).

Regarding response time (Fig 4), neither the main effects of cognitive load nor regulation strategy type were significant. However, the interaction between them was significant (*F*_(2, 75)_ = 15.49, *p* < .001, *η*^2^ = 0.29), indicating that the effect of emotion regulation strategies on processing speed differed depending on cognitive load conditions and providing partial support for H3 from the perspective of response efficiency. Simple main effects analyses of emotion regulation strategy within each level of cognitive load, with Bonferroni adjustments for multiple comparisons, revealed that the free viewing group had significantly longer response time compared to the cognitive reappraisal group in high cognitive load (*MD* = 2324.09, *SE* = 766.18, *p =* .003). In the low cognitive load, the expressive suppression group had a significantly shorter response time compared to both the cognitive reappraisal group (*MD* = 2532.29, *SE* = 714.35, *p* < .001) and the free viewing group (*MD* = 1864.20, *SE* = 732.36, *p =* .013). Simple main effect analyses were conducted within each cognitive load condition to examine differences among emotion regulation strategies. These analyses allow for within-load comparisons but do not directly test differences across load conditions.

### The effects of regulating different emotions on convergent thinking tasks under different cognitive loads

This experiment induced two distinct emotions (anger and anxiety) and employed a 3 (regulation strategy type: cognitive reappraisal, expressive suppression, and free viewing) × 2 (emotion type: anger, anxiety) mixed-design ANOVA under two different cognitive load conditions to examine the effects of regulation strategy type and emotion type on CCRA tasks.

As depicted in Table 3, regardless of the high/low cognitive load condition, the main effect of emotion type was significant. Specifically, participants scored significantly higher on CCRA tasks under anger emotion compared to anxiety emotion (high: *F*_(1, 75)_ = 27.56, *p* < .001, *η*^2^ = 0.27; low: *F*_(1, 75)_ = 47.97, *p* < .001, *η*^2^ = 0.39). However, there was no significant interaction between emotion type and regulation strategy type under different cognitive load conditions (*F*_(1, 75)_ = 1.44, *p =* .243).

**Table 3:** The effects of emotion regulation strategy and emotion type on CCRA task scores under different cognitive loads.

Emotion regulation strategy	High load		Low load	
	Angry	Anxiety	Angry	Anxiety
**Cognitive reappraisal (*n* = 29)**	6.24 ± 2.08	4.48 ± 1.72	6.28 ± 2.21	4.62 ± 1.99
**Expressive suppression (*n* = 23)**	5.87 ± 1.66	5.09 ± 1.62	6.91 ± 1.88	4.91 ± 1.65
**Free viewing (*n* = 26)**	6.50 ± 2.04	4.62 ± 1.72	6.58 ± 2.17	4.58 ± 1.65

*Note.* Values are presented as means ± standard deviations (*SD*).

In terms of response time (Table 4), under high cognitive load conditions, neither the main effects of emotion type and regulation strategy type nor their interaction were significant (all *ps* > .05), indicating that response speed did not differ significantly across emotional states or regulation strategies.

**Table 4:** The effects of regulating different emotions on the response time of CCRA tasks under different cognitive loads.

Emotion regulation strategy	High load		Low load	
	Angry	Anxiety	Angry	Anxiety
**Cognitive reappraisal (*n* = 29)**	13325.26 ± 3446.54	13199.64 ± 3543.93	11891.83 ± 3436.64	11272.52 ± 3494.08
**Expressive suppression (*n* = 23)**	12610.79 ± 3203.30	11995.74 ± 2502.51	11487.21 ± 2644.76	9973.14 ± 2424.11
**Free viewing (*n* = 26)**	14540.33 ± 4126.74	13272.20 ± 3548.26	13783.09 ± 4191.15	11404.84 ± 3445.08

*Note.* Values are presented as means ± standard deviations (*SD*). Response time is measured in milliseconds.

Under low cognitive load conditions, a significant main effect of emotion type was observed (*F*_(1, 75)_ = 8.54, *p* = .005, *η*^2^ = .10), with participants responding faster to anxiety-related stimuli than anger-related stimuli (*MD* = 1504.15, *SE* = 514.83, *p* = .005 < .01). The main effect of regulation strategy type was also significant (*F*_(1, 75)_ = 3.47, *p* = .036, *η*^2^ = .09). Specifically, participants in the expressive suppression group responded faster than those in the free viewing group (*MD* = 1864.20, *SE* = 710.15, *p* = .010). However, the interaction between emotion type and regulation strategy type was not significant (*F*_(2, 75)_ = 1.04, *p* > .05, *η*^2^ = .03), suggesting that the effect of regulation strategy on response time did not significantly differ between anger and anxiety conditions.

## Discussion

Guided by the emotion regulation flexibility framework, this study examined whether the effectiveness of emotion regulation strategies depends on cognitive load as an important contextual demand. Overall, the findings supported H1 and H2, indicating that emotion regulation strategies effectively influenced emotional states and that cognitive load affected convergent thinking performance. H3 was partially supported, as the interaction between emotion regulation strategies and cognitive load was observed in response efficiency but not in convergent thinking scores.

This experiment incorporated both emotion regulation strategies and tasks of varying cognitive loads. Participants were required to complete the CCRA task, which allowed examination of how these strategies influence convergent thinking under different load conditions. To better interpret the findings, it is useful to consider them through the lens of the distinction between performance speed and accuracy [60]. Accuracy in convergent thinking tasks such as the CCRA reflects the successful retrieval and integration of relevant knowledge, indicating effective problem-solving under the given conditions [51]. In contrast, response time reflects the efficiency of cognitive processing, including attentional control, working memory updating, and inhibition of irrelevant information [61]. Discrepancies between accuracy and speed outcomes suggest that an emotion regulation strategy may facilitate faster information processing without necessarily improving the correctness of responses, or vice versa. For example, under high cognitive load, a reduction in response time without a corresponding increase in accuracy may indicate a shift toward more heuristic or less effortful processing. Conversely, improvements in accuracy without faster responses could reflect deeper, more deliberate problem-solving at the expense of speed. This distinction provides a nuanced framework for understanding how cognitive load and emotion regulation interact to shape convergent thinking performance. Building on this perspective, further analysis was conducted on the impact of regulating different types of emotions under varying cognitive loads on convergent thinking tasks.

### Analysis of the effects of emotion regulation strategies

The results showed that, under both high and low cognitive load conditions, emotion ratings followed the trend of cognitive reappraisal > expressive suppression > free viewing (higher scores indicating more positive emotions). This finding suggests that both classical emotion regulation strategies—cognitive reappraisal and expressive suppression—were effective in reducing negative emotions, with cognitive reappraisal demonstrating a significantly stronger regulatory effect. These results are consistent with H1. An additional theoretical consideration concerns whether the effectiveness of emotion regulation may represent a potential mechanism linking regulation strategy to convergent thinking performance [62]. In the present study, emotion ratings indicated that cognitive reappraisal was associated with relatively more positive emotional outcomes compared to expressive suppression and free viewing. It is therefore plausible that the degree to which emotions were successfully regulated may have contributed to performance differences across strategies [62,63].

Cognitive reappraisal involves reinterpreting the meaning of a situation to alter its emotional impact, particularly when facing negative stimuli [15]. The present findings are in line with previous research [64], further confirming the advantage of cognitive reappraisal in emotion regulation. Ertl, Hildebrandt [15] found that, compared with maintaining negative emotions, participants engaging in cognitive reappraisal exhibited a significant increase in frontal theta activity (around 4 Hz at the Fz electrode), which was positively correlated with self-reported success in applying the strategy. This indicates that reappraisal involves enhanced cognitive control processes.

The superiority of cognitive reappraisal over expressive suppression has also been demonstrated in applied contexts. For instance, individuals who habitually use reappraisal report higher job satisfaction than those relying on suppression [65]. Similarly, Yeung and Wong [64] observed that employees who used reappraisal, regardless of age, experienced less negative affect and perceived stress in daily life. However, it is important to note that reappraisal is not always adaptive, and its effectiveness depends on situational controllability. Troy, Shallcross [66] found that when stressors were uncontrollable, higher reappraisal ability predicted lower depression. However, when stressors were controllable, greater reappraisal use was associated with higher depression. This suggests that the adaptiveness of cognitive reappraisal is context-dependent.

### The impact of emotion regulation strategies on convergent thinking tasks under different cognitive loads

The results indicate that performance on convergent thinking tasks was significantly better under low cognitive load compared to high cognitive load, suggesting that increased cognitive load impairs convergent thinking in college students. This finding supports H2, which proposed that limited cognitive resources constrain creative problem solving. Furthermore, when scores were similar, individuals who solved problems more rapidly exhibited enhanced task performance to a certain extent, consistent with findings by Zhu, Zhao [62] that cognitive processing speed can influence task outcomes. Although the cognitive load × regulation strategy interaction on convergent thinking scores was not significant, this result suggests that different emotion regulation strategies did not significantly influence final task accuracy across cognitive load conditions. Therefore, H3 was not supported for accuracy scores. However, from the perspective of the Emotion Regulation Flexibility Framework, the effectiveness of a regulation strategy may depend on the specific performance indicator and contextual demands. The significant interaction observed in response time indicates that regulation strategies may influence processing efficiency rather than final task accuracy. Specifically, the cognitive reappraisal group showed faster responses than the free viewing group under high cognitive load, whereas the expressive suppression group showed faster responses than both the cognitive reappraisal and free viewing groups under low cognitive load. This pattern suggests that strategy–context fit may be reflected more clearly in the speed of convergent thinking than in accuracy outcomes.

Under low cognitive load, the participants showed better performance in convergent thinking, which is similar to the findings of Bi, Zheng [67]. In their study, Bi, Zheng [67] explored the effects of cognitive load on prospective memory with 31 college students as participants. In terms of cognitive load, the participants had significantly higher accuracy in completing the Stroop task under low cognitive load compared to high cognitive load (97.3% vs. 95.7%). Similarly, a study conducted by Park and Brünken [68], in which 30 participants completed a rhythm recognition task under varying cognitive loads, found that under low cognitive load, participants achieved significantly higher accuracy in rhythm recognition, and learning rhythm recognition under low cognitive load notably improved recognition accuracy. When individuals need to complete other cognitive tasks while performing convergent thinking tasks, these two types of tasks engage in intense competition, collectively exhausting limited cognitive resources [69]. The greater the cognitive load required by a task, the more attention resources individuals will invest. As a result, they will have fewer attention resources available for completing convergent thinking tasks. However, under low cognitive load, individuals have sufficient attentional resources to complete convergent thinking tasks, leading to significantly higher scores [70].

In research pertaining to cognitive load, response time often serves as an indicator for examining the impact of various cognitive loads on cognitive activities [67]. When individuals encounter high cognitive load, the persistence of negative emotions notably extends the time required for problem-solving. Intense negative emotions may prompt individuals to exert efforts in self-regulation to adapt to their current environment [51,71]. At the same time, such heightened negative emotions might motivate individuals to strive to avoid failure, thereby increasing their willingness to invest more time in solving problems [39]. However, it’s important to note that there are discrepancies between the findings of this experiment and those of other studies. For instance, Zhang, Leng [72] conducted a study involving 57 participants to investigate the influence of emotional experiences and cognitive load on working memory. Their results revealed that, in high cognitive load tasks, participants exhibited longer response times when experiencing pleasant emotions, whereas response times were significantly shorter when experiencing aversive emotions, as compared to neutral emotions. The disparity in results may be attributed to the fact that this experiment elicited anxiety and anger emotions in participants. Despite both being negative emotions, anxiety, and anger possess fundamentally distinct characteristics that can lead to variations in outcomes. These findings collectively suggest that the type and intensity of negative emotions, combined with cognitive load, play a complex role in influencing cognitive task performance, which is reflected in our study’s results.

Overall, the findings suggested that higher cognitive load may hinder convergent thinking performance. Convergent thinking tended to decline under high cognitive load, indicating that increased task demands could constrain creative problem solving. Under low cognitive load, expressive suppression was linked to faster responses, implying a possible efficiency advantage when cognitive resources are sufficient. This finding is consistent with a study by Wu, Guo [73], in which 101 participants were divided into expressive suppression, cognitive reappraisal, and control groups to investigate the impact of emotion regulation strategies on working memory tasks of varying difficulty levels. The results indicated no discernible difference between the two strategies in the low difficulty working memory task. However, in tasks of medium and high difficulty, the cognitive reappraisal group exhibited significantly higher accuracy rates compared to the expressive suppression group. In this study, we observed that under low cognitive load, the expressive suppression group demonstrated faster response times, implying that individuals can employ expressive suppression to regulate negative emotions and expedite response times when solving convergent thinking problems.

These patterns can be understood within Gross’s process model of emotion regulation [10] and cognitive resource theories [74]. Cognitive reappraisal, being an antecedent-focused strategy, demands substantial working memory and attentional control resources to reinterpret emotional stimuli [29]. From a resource competition perspective, one might predict that engaging in a cognitively demanding strategy such as reappraisal under high cognitive load would place individuals at an additional disadvantage [38]. However, emotion regulation may also reduce the cognitive costs associated with intrusive negative affect. Under high cognitive load, negative emotions may further disrupt task-relevant attention and consume limited cognitive resources [75]. Although reappraisal requires initial cognitive investment, it may mitigate sustained emotional interference, thereby stabilizing attentional control and supporting task performance [46]. Expressive suppression, in contrast, is a response-focused strategy that primarily targets behavioral expression and may require fewer cognitive resources in low-load situations [46]. In low-load contexts, suppression may reduce overt emotional expression with relatively lower cognitive demands, potentially allowing more resources for rapid information processing [76]. This framework may help explain the observed descriptive patterns (i.e., higher mean scores for cognitive reappraisal under high load and for expressive suppression under low load). Nevertheless, given the non-significant interaction, these interpretations should be considered tentative.

Another theoretical consideration concerns individuals’ habitual use of emotion regulation strategies. Previous research suggests that cognitive reappraisal is more frequently and habitually used in daily life compared to expressive suppression [8]. Although no significant group differences were observed in ERQ scores in the present study, it is possible that participants were generally more familiar or practiced with reappraisal as a regulation strategy. Habitual use may facilitate more efficient or automatic implementation of the strategy, particularly under conditions of cognitive constraint [77]. From this perspective, the descriptive tendency for cognitive reappraisal to be associated with relatively higher scores under high cognitive load may partly reflect greater strategy familiarity or ease of deployment [45]. However, given the non-significant interaction observed in the present study, this interpretation remains speculative and warrants further investigation in future research.

### The impact of regulating different emotions on convergent thinking tasks under different cognitive loads

This experiment induced two emotions (anger and anxiety) in participants, leading to varied performance under different negative emotions. In summary, irrespective of the cognitive load level (high or low), participants’ scores on convergent thinking tasks were significantly higher when experiencing anger as compared to anxiety. Under low cognitive load, the response time of participants under anxiety emotion was significantly faster than under anger emotion.

This experiment revealed a facilitative effect of anger on convergent thinking tasks. While anger is often associated with impulsivity and can potentially induce risky behavior [78], it also possesses a dual nature, exerting positive effects on cognitive activities. As the results of this experiment demonstrate, individuals experiencing anger emotion perform better in convergent thinking tasks compared to those experiencing anxiety emotion. Anxiety is generally considered a negative emotion, and cognitive models suggest that it can lead to various biases in information processing, such as attentional biases and interpreting ambiguous information as threatening [79]. Anxiety can affect various aspects of college students’ lives and studies. For instance, in sports activities, anxiety can affect physiological arousal, disrupt attention, create self-doubt, and generate fear of competition, thereby impacting performance [80]. In exams, anxiety can lead to “mind blanking” and affect test scores. College students often encounter various negative emotions during the process of completing learning tasks, such as anxiety, anger, frustration, and worry [81]. Regarding anxiety and anger, college students perceive anxiety experiences as stronger in these tasks, and the intensity of negative experiences increases with task difficulty. This study suggests that anger emotion can facilitate performance in convergent thinking tasks. Therefore, although college students may experience negative emotions when engaging in moderately difficult tasks, effectively utilizing these negative emotions may have positive effects on task performance [81].

In anxiety research, response time is often deemed a reliable indicator [62,79]. This study found that under low cognitive load tasks, participants experiencing anxiety exhibited significantly shorter response times. This result may reflect the facilitating effect of moderate anxiety on alertness and information processing speed, consistent with arousal theories suggesting that negative emotions can enhance performance when task demands are low [82,83]. Unlike anger, which is an approach-oriented emotion promoting deeper problem-focused processing, anxiety may heighten vigilance and readiness to respond, leading to faster reactions in simple tasks. This finding diverges from Brown, Eley [79], who reported a negative correlation between anxiety and processing speed in face recognition. The discrepancy may be due to methodological differences: Brown, Eley [79] measured trait anxiety through questionnaires in children, whereas the present study induced transient anxiety in college students. This difference may be due to the fact that Brown et al. measured participants’ anxiety levels using questionnaires, while this experiment induced immediate anxiety emotions in the participants. Additionally, Brown, Eley [79] studied children, while this experiment focused on college students. These results suggest that the impact of anxiety on cognitive performance is sensitive to both the nature of the task and the type of anxiety measured, highlighting the dual role of anxiety in facilitating rapid responses while potentially impairing complex problem-solving. Moreover, the present findings should be interpreted within the Chinese cultural context. Previous cross-cultural research has suggested that cultural differences may influence individuals’ psychological characteristics and behavioral patterns [84], and cultural norms regarding emotional expression may further affect the use and outcomes of different emotion regulation strategies. Therefore, future studies involving participants from diverse cultural backgrounds are needed to examine the cross-cultural generalizability of these findings.

### Limitations and future directions

There are some limitations to this study. First, the assessment and implementation of emotion regulation strategies may warrant further consideration. Although participants reported their use of the instructed strategies, self-report measures may not fully capture whether participants exclusively relied on a specific strategy, and some degree of strategy overlap across conditions may have occurred. Future research could employ more fine-grained assessments to evaluate strategy use and examine the role of regulation effectiveness in explaining individual differences in task performance. Second, this study focused primarily on the regulation of specific negative emotions (anger and anxiety). Future studies could examine whether the regulation of other negative emotions (e.g., sadness and fear), positive emotions, or more complex emotions produces different effects on convergent thinking. Third, the interpretation of differences between high and low cognitive load conditions should be made with caution, as the simple main effect analyses were conducted within each load level separately rather than in a single integrated model including both load conditions. Although this approach allowed us to focus on strategy effects under distinct cognitive constraints, it does not permit direct statistical comparison across load conditions. Fourth, the relatively modest sample size (*N* = 78) may have limited the statistical power to detect smaller effects or interaction patterns. Moreover, the participants in this study were Chinese undergraduate students within a relatively narrow age range, which may limit the generalizability of the findings to individuals from other developmental stages and cultural backgrounds. Cultural norms regarding emotional expression and regulation may influence the use and effectiveness of different emotion regulation strategies. Future studies should include larger and more diverse samples across multiple universities and cultural contexts to enhance the robustness and external validity of the results. Finally, data collection occurred toward the end of the COVID-19 pandemic, which may have affected participant recruitment and contributed to gender imbalance. Future studies should recruit more gender-balanced samples to enhance generalizability. Furthermore, although the experimental design of the present study provides evidence regarding the immediate effects of emotion regulation strategies and cognitive load on convergent thinking, longitudinal approaches would be valuable for examining how emotion regulation abilities and creative cognition change over time. Future longitudinal studies may provide a more comprehensive understanding of the dynamic relationships among emotion regulation processes, cognitive demands, and creative cognition.

## Conclusion

This study focuses on convergent thinking in creative thinking and examines the impact of regulating emotion under different cognitive loads on convergent thinking tasks. In terms of response time, a significant interaction emerged between cognitive load and regulation strategy. Under high cognitive load, the free-viewing group showed longer response times than the cognitive reappraisal group, whereas under low cognitive load, the expressive suppression group showed shorter response times than both the cognitive reappraisal and free-viewing groups. Moreover, this study elicited distinct emotions in participants, revealing that irrespective of cognitive load levels, anger emotion consistently yielded significantly higher scores in convergent thinking tasks. Interestingly, under low cognitive load, participants exhibited notably quicker response times when experiencing anxiety emotions as opposed to anger emotions. The results of this study can provide meaningful suggestions for college students to promote the performance of convergent thinking by regulating emotions.

These findings provide practical implications for college students and educators. For students, developing the ability to regulate emotions, particularly through cognitive reappraisal, may help maintain cognitive flexibility and improve problem-solving efficiency under stress. For educators, integrating emotion regulation training into classroom instruction could foster students’ emotional awareness, adaptive thinking, and creative potential in demanding learning environments.

## Supporting information

S1 File Data. (XLSX)

## References

[1] Besançon M, Lubart T. Differences in the development of creative competencies in children schooled in diverse learning environments. Learn Indiv Diff. 2008;18(4):381–9. doi: 10.1016/j.lindif.2007.11.009

[2] Cortes RA, Weinberger AB, Daker RJ, Green AE. Re-examining prominent measures of divergent and convergent creativity. Curr Opin Behav Sci. 2019;27:90–3. doi: 10.1016/j.cobeha.2018.09.017

[3] de Rooij A, Vromans RD. The (dis)pleasures of creativity: spontaneous eye blink rate during divergent and convergent thinking depends on individual differences in positive and negative affect. J Creat Behav. 2018;54(2):436–52. doi: 10.1002/jocb.379

[4] Eon Duval P, Frick A, Denervaud S. Divergent and convergent thinking across the schoolyears: a dynamic perspective on creativity development. J Creat Behav. 2022;57(2):186–98. doi: 10.1002/jocb.569

[5] Guilford JP. Creativity: yesterday, today and tomorrow. J Creat Behav. 1967;1(1):3–14. doi: 10.1002/j.2162-6057.1967.tb00002.x

[6] Zhao Y, Yuan Y, Shen W, Zhu C, Liu D. The relationships between bilingual learning, willingness to study abroad and convergent creativity. PeerJ. 2019;7:e7776. doi: 10.7717/peerj.7776 31579628 PMC6766371

[7] Karthikeyan K, Vsudevan A, Sowmya K, Jamil NA, Mohamad Hanefar SB. AI-enabled creativity: effects on divergent and convergent thinking among higher education students. Int J Eng Ped. 2025;15(7):52–68. doi: 10.3991/ijep.v15i7.59051

[8] Shen W, Luo J, Liu C, Yuan Y. New advances in the neural correlates of insight: a decade in review of the insightful brain. Chin Sci Bull. 2013;58:1497–511.

[9] Gross JJ, John OP. Individual differences in two emotion regulation processes: implications for affect, relationships, and well-being. J Pers Soc Psychol. 2003;85(2):348–62. doi: 10.1037/0022-3514.85.2.348 12916575

[10] Gross JJ. The emerging field of emotion regulation: an integrative review. Rev General Psychol. 1998;2(3):271–99. doi: 10.1037/1089-2680.2.3.271

[11] Zou M, Liu B, Ren L, Mu D, He Y, Yin M, et al. The association between aspects of expressive suppression emotion regulation strategy and rumination traits: a network analysis approach. BMC Psychol. 2024;12(1):501. doi: 10.1186/s40359-024-01993-2 39334290 PMC11438175

[12] Bebko GM, Franconeri SL, Ochsner KN, Chiao JY. Attentional deployment is not necessary for successful emotion regulation via cognitive reappraisal or expressive suppression. Emotion. 2014;14(3):504–12. doi: 10.1037/a0035459 24512245

[13] Wang YM, Chen J, Han BY. The effects of cognitive reappraisal and expressive suppression on memory of emotional pictures. Front Psychol. 2017;8:1921. 29163298 10.3389/fpsyg.2017.01921 PMC5672560

[14] Zhang X j, Liu X, Qu X. How team task conflict influences individual creativity: a cross-level mediated moderation model. Forecasting. 2016;35(1):22–7.

[15] Ertl M, Hildebrandt M, Ourina K, Leicht G, Mulert C. Emotion regulation by cognitive reappraisal - the role of frontal theta oscillations. Neuroimage. 2013;81:412–21. doi: 10.1016/j.neuroimage.2013.05.044 23689018

[16] Toscano F, Giusino D, Diana R, Rahimi Pordanjani T. The role of emotional regulation in the relationship between nurses’ creative style and innovation behaviors: a cross-sectional study. Nurs Rep. 2023;13(2):811–22. doi: 10.3390/nursrep13020071 37368338 PMC10301246

[17] Zhao Y. The influence of emotion regulation strategies on college students’ convergent creativity and its neural mechanism. Soochow University; 2021.

[18] Subramaniam K, Kounios J, Parrish TB, Jung-Beeman M. A brain mechanism for facilitation of insight by positive affect. J Cogn Neurosci. 2009;21(3):415–32. doi: 10.1162/jocn.2009.21057 18578603

[19] Wang M, Takahashi Y, Cheung C. The interplay between negative activating emotions, family expressiveness, and gender: implications for creativity. J Creat Behav. 2024;58(4):561–76. doi: 10.1002/jocb.684

[20] Baas M, De Dreu CKW, Nijstad BA. When prevention promotes creativity: the role of mood, regulatory focus, and regulatory closure. J Pers Soc Psychol. 2011;100(5):794–809. doi: 10.1037/a0022981 21381857

[21] Redifer JL, Bae CL, DeBusk-Lane M. Implicit theories, working memory, and cognitive load: impacts on creative thinking. Sage Open. 2019;9(1). doi: 10.1177/2158244019835919

[22] Yu W, Qin X, Li M, Xue X. Cognitive load and creativity of knowledge workers: a diary study. Curr Psychol. 2023;43(15):13386–401. doi: 10.1007/s12144-023-05395-2

[23] Hernandez Sibo IP, Gomez Celis DA, Liou S. Exploring the landscape of cognitive load in creative thinking: a systematic literature review. Educ Psychol Rev. 2024;36(1). doi: 10.1007/s10648-024-09866-1

[24] Pham TP, Sanocki T. Human attention restoration, flow, and creativity: a conceptual integration. J Imaging. 2024;10(4):83. doi: 10.3390/jimaging10040083 38667981 PMC11050943

[25] Rodet CS. Does cognitive load affect creativity? An experiment using a divergent thinking task. Econ Lett. 2022;220:110849. doi: 10.1016/j.econlet.2022.110849

[26] Gu Q. The influence of language level on cognitive load in listening and reading comprehension of second language learners. Foreign Lang Educ. 2020;41(3):78–83.

[27] Huang Y, Song X, Ye Q. Mind wandering and the incubation effect: investigating the influence of working memory capacity and cognitive load on divergent thinking. Think Skills Creat. 2024;52:101499. doi: 10.1016/j.tsc.2024.101499

[28] Niermeyer MA, Ziemnik RE, Franchow EI, Barron CA, Suchy Y. Greater naturally occurring expressive suppression is associated with poorer executive functioning and motor-sequence learning among older adults. J Clin Exp Neuropsychol. 2019;41(2):118–32. doi: 10.1080/13803395.2018.1502257 30102116

[29] Ochsner KN, Gross JJ. The cognitive control of emotion. Trends Cogn Sci. 2005;9(5):242–9. 15866151 10.1016/j.tics.2005.03.010

[30] Nusbaum EC, Silvia PJ. Are intelligence and creativity really so different?☆Fluid intelligence, executive processes, and strategy use in divergent thinking. Intelligence. 2011;39(1):36–45. doi: 10.1016/j.intell.2010.11.002

[31] Asberg K. Hostility/anger as a mediator between college students’ emotion regulation abilities and symptoms of depression, social anxiety, and generalized anxiety. Emotions and their influence on our personal, interpersonal and social experiences. Routledge; 2018. pp. 178–99. 10.1080/00223980.2012.715601 24003591

[32] Rosenthal BS, Schreiner AC. Prevalence of psychological symptoms among undergraduate students in an ethnically diverse urban public college. J Am Coll Health. 2000;49(1):12–8. doi: 10.1080/07448480009596277 10967879

[33] Lindert J, Paul KC, Lachman ME, Ritz B, Seeman TE. Depression-, anxiety-, and anger and cognitive functions: findings from a longitudinal prospective study. Front Psychiatry. 2021;12:665742. doi: 10.3389/fpsyt.2021.665742 34421666 PMC8377351

[34] Perchtold-Stefan CM, Fink A, Rominger C, Papousek I. Failure to reappraise: malevolent creativity is linked to revenge ideation and impaired reappraisal inventiveness in the face of stressful, anger-eliciting events. Anxiety Stress Coping. 2021;34(4):437–49. doi: 10.1080/10615806.2021.1918682 33899626 PMC8367047

[35] Carver CS, Harmon-Jones E. Anger is an approach-related affect: evidence and implications. Psychol Bull. 2009;135(2):183–204. doi: 10.1037/a0013965 19254075

[36] Baas M, De Dreu CK, Nijstad BA. A meta-analysis of 25 years of mood-creativity research: Hedonic tone, activation, or regulatory focus? Psychol Bull. 2008;134(6):779. 18954157 10.1037/a0012815

[37] Berggren N, Derakshan N. Attentional control deficits in trait anxiety: why you see them and why you don’t. Biol Psychol. 2013;92(3):440–6. doi: 10.1016/j.biopsycho.2012.03.007 22465045

[38] Gan S, Yang J, Chen X, Zhang X, Yang Y. High working memory load impairs the effect of cognitive reappraisal on emotional response: Evidence from an event-related potential study. Neurosci Lett. 2017;639:126–31. doi: 10.1016/j.neulet.2016.12.069 28041962

[39] Plass JL, Kalyuga S. Four ways of considering emotion in cognitive load theory. Educ Psychol Rev. 2019;31(2):339–59. doi: 10.1007/s10648-019-09473-5

[40] Bonanno G, Burton C. Regulatory Flexibility. Perspect Psychol Sci. 2013;8:591–612. doi: 10.1177/1745691613504116 26173226

[41] Kaur K, Lohani M, Williams P, Asnaani A. A person-specific emotion regulation flexibility framework: taking an integrative approach. Emot Rev. 2025;17(4):229–46. doi: 10.1177/17540739251323517

[42] Specker P, Sheppes G, Nickerson A. Does emotion regulation flexibility work? Investigating the effectiveness of regulatory selection flexibility in managing negative affect. Soc Psychol Pers Sci. 2023;15(5):561–9. doi: 10.1177/19485506231189002

[43] Dougherty EN, Bottera AR, Haedt-Matt AA, Wildes JE. Reconceptualizing emotion regulation and coping strategy usage in eating disorders research: The utility of a regulatory flexibility framework. Int J Eat Disord. 2023;56(10):1835–41. doi: 10.1002/eat.24027 37465948 PMC10592414

[44] Zhou S, Wu Y, Xu X. Linking cognitive reappraisal and expressive suppression to mindfulness: a three-level meta-analysis. Int J Environ Res Public Health. 2023;20(2):1241. doi: 10.3390/ijerph20021241 36673984 PMC9859341

[45] Brockbank RB, Feldon DF. Cognitive reappraisal: the bridge between cognitive load and emotion. Educ Sci. 2024;14(8):870. doi: 10.3390/educsci14080870

[46] Cutuli D. Cognitive reappraisal and expressive suppression strategies role in the emotion regulation: an overview on their modulatory effects and neural correlates. Front Syst Neurosci. 2014;8:175. doi: 10.3389/fnsys.2014.00175 25285072 PMC4168764

[47] Zhao Y, Yin M, Zhu C, Tan C, Hu S, Liu D. Can situations awaken emotions? The compilation and evaluation of the Emotional Situation Sentence System (ESSS). PLoS One. 2021;16(7):e0252671. doi: 10.1371/journal.pone.0252671 34280194 PMC8289053

[48] Shen W, Yuan Y, Liu C, Baoshu YI, Dou K. The development and validity of a chinese version of the compound remote associates test. Am J Psychol. 2016;129:245–58. doi: 10.5406/amerjpsyc.129.3.0245 29558590

[49] Benoit ID, Miller EG. Enhancing creativity perception through fear. J Bus Res. 2022;139:1084–98. doi: 10.1016/j.jbusres.2021.10.051

[50] Watson D, Clark LA, Tellegen A. Development and validation of brief measures of positive and negative affect: the PANAS scales. J Pers Soc Psychol. 1988;54(6):1063–70. doi: 10.1037//0022-3514.54.6.1063 3397865

[51] Shen W, Zhao Y, Hommel B, Yuan Y, Zhang Y, Liu Z, et al. The impact of spontaneous and induced mood states on problem solving and memory. Think Skills Creat. 2019;32:66–74. doi: 10.1016/j.tsc.2019.03.002

[52] Leung AK, Kwan L, Liou S, Chiu C, Qiu L, Yong JC. The role of instrumental emotion regulation in the emotions-creativity link: how worries render neurotic individuals more creative. In: Proceedings of the 9th ACM Conference on Creativity & Cognition. 2013.

[53] Macrae CN, Hewstone M, Griffiths RJ. Processing load and memory for stereotype‐based information. Euro J Soc Psychol. 1993;23(1):77–87. doi: 10.1002/ejsp.2420230107

[54] Cheng L, He Y, OuYang H, Li G, Li H. The modulation of emotional response to fearful pictures: A comparison between cognitive reappraisal and expressive suppression—An ERP study. J Psychol Sci. 2011;34:925–30.

[55] Yuan J, Long Q, Ding N, Lou Y, Liu Y, Yang J. Suppression dampens unpleasant emotion faster than reappraisal: Neural dynamics in a Chinese sample. Sci China Life Sci. 2015;58(5):480–91. doi: 10.1007/s11427-014-4739-6 25316046

[56] Shen W, Yuan Y, Liu C, Luo J. In search of the ‘Aha!’ experience: Elucidating the emotionality of insight problem‐solving. Br J Psychol. 2016;107(2):281–98. 26184903 10.1111/bjop.12142

[57] Norouzian R, Plonsky L. Eta- and partial eta-squared in L2 research: a cautionary review and guide to more appropriate usage. Second Lang Res. 2017;34(2):257–71. doi: 10.1177/0267658316684904

[58] Brydges CR. Effect size guidelines, sample size calculations, and statistical power in gerontology. Innov Aging. 2019;3(4):igz036. doi: 10.1093/geroni/igz036 31528719 PMC6736231

[59] Gilbert DT, Hixon JG. The trouble of thinking: Activation and application of stereotypic beliefs. J Pers Soc Psychol. 1991;60(4):509–17. doi: 10.1037/0022-3514.60.4.509

[60] Bali KK, Gupta A, Ong YS, Tan PS. Cognizant multitasking in multiobjective multifactorial evolution: MO-MFEA-II. IEEE Trans Cybern. 2020;51(4):1784–96. 10.1109/TCYB.2020.2981733 32324586

[61] Bieth T, Ovando-Tellez M, Lopez-Persem A, Garcin B, Hugueville L, Lehongre K, et al. Time course of EEG power during creative problem-solving with insight or remote thinking. Hum Brain Mapp. 2024;45(1):e26547. doi: 10.1002/hbm.26547 38060194 PMC10789201

[62] Zhu C, Zhao X, Han X, Wang Y, Liu D, Luo W. Estimation strategy selection is modulated by snapshot emotional priming, but not math anxiety. Int J Environ Res Public Health. 2022;19(16):10268. doi: 10.3390/ijerph191610268 36011903 PMC9408359

[63] Mohammed A-R, Kosonogov V, Lyusin D. Expressive suppression versus cognitive reappraisal: Effects on self-report and peripheral psychophysiology. Int J Psychophysiol. 2021;167:30–7. doi: 10.1016/j.ijpsycho.2021.06.007 34157337

[64] Yeung DY, Wong S. Effects of cognitive reappraisal and expressive suppression on daily work-related outcomes: Comparison between younger and older Chinese workers. Int J Psychol. 2020;55(6):983–94. doi: 10.1002/ijop.12661 32017064

[65] Cossette M, Hess U. Service with style and smile. How and why employees are performing emotional labour? Eur Rev Appl Psychol. 2015;65(2):71–82. doi: 10.1016/j.erap.2015.02.001

[66] Troy AS, Shallcross AJ, Mauss IB. A person-by-situation approach to emotion regulation: cognitive reappraisal can either help or hurt, depending on the context. Psychol Sci. 2013;24(12):2505–14. doi: 10.1177/0956797613496434 24145331

[67] Bi R, Zheng X, Sun M, Wei P, Wang Y. The impact of absolute importance and cognitive load on event-based prospective memory. J Psychol Sci. 2019;42(1):29.

[68] Park B, Brünken R. The rhythm method: a new method for measuring cognitive load—an experimental dual‐task study. Appl Cogn Psychol. 2014;29(2):232–43. doi: 10.1002/acp.3100

[69] Shang L, Little DR, Webb ME, Eidels A, Yang C-T. The workload capacity of semantic search in convergent thinking. J Exp Psychol Gen. 2021;150(11):2230–45. doi: 10.1037/xge0001045 34498907

[70] Zhang J, Zhang M. An experimental study of effects of tension and cognitive load on prospective memory. J Nanjing Inst Technol (Soc Sci Ed). 2011;11(4):54–8.

[71] Friedman RS, Förster J. The effects of approach and avoidance motor actions on the elements of creative insight. J Pers Soc Psychol. 2000;79(4):477–92. doi: 10.1037//0022-3514.79.4.477 11045734

[72] Zhang P, Leng Y, Lu J. The empirical research on the influence of emotional experience and cognitive load on the working memory. Psychol Explor. 2017;307:17–22.

[73] Wu P, Guo S, He W. The influence of emotion regulation on the cognitive effect of negative meta-stereotype activation. Psychol Explor. 2019;39(4):368–73.

[74] Norman DA, Bobrow DG. On data-limited and resource-limited processes. Cogn Psychol. 1975;7(1):44–64. doi: 10.1016/0010-0285(75)90004-3

[75] Domic-Siede M, Guzmán-González M, Sánchez-Corzo A, Granillo L, Salazar C, Silva M, et al. Pupil responses to emotion regulation strategies: the role of attachment orientations. Cogn Emot. 2026;40(2):404–21. doi: 10.1080/02699931.2025.2512886 40472472

[76] Scheffel C, Graupner S-T, Gärtner A, Zerna J, Strobel A, Dörfel D. Effort beats effectiveness in emotion regulation choice: differences between suppression and distancing in subjective and physiological measures. Psychophysiology. 2021;58(11):e13908. doi: 10.1111/psyp.13908 34310724

[77] Riepenhausen A, Wackerhagen C, Reppmann ZC, Deter H-C, Kalisch R, Veer IM, et al. Positive cognitive reappraisal in stress resilience, mental health, and well-being: a comprehensive systematic review. Emotion Rev. 2022;14(4):310–31. doi: 10.1177/17540739221114642

[78] Oehl M, Brandenburg S, Huemer AK. Cyclists’ anger experiences in traffic: The cycling anger scale. Transportation research part F: traffic psychology and behaviour. 2019;62:564–74.

[79] Brown HM, Eley TC, Broeren S, Macleod C, Rinck M, Hadwin JA, et al. Psychometric properties of reaction time based experimental paradigms measuring anxiety-related information-processing biases in children. J Anxiety Disord. 2014;28(1):97–107. doi: 10.1016/j.janxdis.2013.11.004 24486916

[80] Ai X, Wang C, Zhang M. The effect of competition state anxiety on volleyball serve performance: the moderating effect of cognitive load. J Xi’an Phys Educ Univ. 2019;4:479–84.

[81] Huang K, Yuan X, Li L, Ye M. Research on users’ negative emotion and related influence factors during collaborative information searching based on affective load theory. Document Inform Knowl. 2020;(1):42–52.

[82] Diamond DM, Campbell AM, Park CR, Halonen J, Zoladz PR. The temporal dynamics model of emotional memory processing: a synthesis on the neurobiological basis of stress-induced amnesia, flashbulb and traumatic memories, and the Yerkes-Dodson law. Neural Plast. 2007;2007:60803. doi: 10.1155/2007/60803 17641736 PMC1906714

[83] Yerkes AW. Review of modifiability of behavior in its relation to the age and sex of the dancing mouse and the relation of strength of stimulus to rapidity of habit-formation. 1910.

[84] Abaie E, Lester D, Kalantar SM. Individual competitiveness, sense of defeat, and organizational commitment: comparisons between Iranian and USA employees. Cross-Cult Commun. 2021;17(2):93–8.

